# Straight-Channel NiO/CeO_2_ Ceramic Reactor Fabricated via Mesh-Assisted Phase Inversion for Catalytic Oxidation of Ventilation Air Methane

**DOI:** 10.3390/ma19091718

**Published:** 2026-04-23

**Authors:** Fangsheng Liu, Enming Shi, Zhiqiang Cao, Xuemei Ou, Fangjun Jin, Dingying Zhou, Zhen Wang, Xinyi Han, Shiru Le, Yeqing Wang

**Affiliations:** 1 School of Materials Science and Physics, China University of Mining and Technology, Xuzhou 221116, China; semhh77@163.com (E.S.); caozq6666@163.com (Z.C.); oxm@cumt.edu.cn (X.O.); jinfj@cumt.edu.cn (F.J.); zdy051010@163.com (D.Z.); wangzhen25@cumt.edu.cn (Z.W.); hhhhxxxyyy111@163.com (X.H.); 2 School of Chemistry and Chemical Engineering, Harbin Institute of Technology, Harbin 150001, China; leshiru@hit.edu.cn

**Keywords:** ventilation air methane, catalytic combustion, NiO/CeO_2_ ceramic reactor, mesh-assisted phase inversion, straight-channel structure

## Abstract

**Highlights:**

- A straight-channel NiO/CeO_2_ ceramic reactor was fabricated via one-step mesh-assisted phase inversion, simplifying the preparation of structured catalysts.
- The reactor, featuring efficient mass transfer, achieves direct and efficient catalytic oxidation of ventilation air methane, and exhibits excellent thermal shock resistance and structural robustness under actual operating conditions.
- Optimal parameters (60 wt% NiO, 1300 °C) based on balancing catalytic activity and mechanical strength were obtained, achieving long-term stable operation.

**Abstract:**

Ventilation air methane (VAM) has an extremely low concentration, making its abatement exceptionally challenging. Catalytic oxidation offers a promising route for VAM treatment, but industrial application requires integrated catalysts with high activity and efficient mass transfer. In this study, a novel straight-channel NiO/CeO_2_ ceramic reactor was fabricated via mesh-assisted phase inversion, with NiO content systematically optimized to screen the optimal ratio. The 60 wt% NiO was the optimal composition, exhibiting excellent VAM oxidation performance. Brunauer–Emmett–Teller (BET) analysis confirmed that this optimal ratio yielded the largest specific surface area. Furthermore, H_2_-temperature-programmed reduction (H_2_-TPR) and X-ray photoelectron spectroscopy (XPS) confirmed that this optimal ratio facilitated the formation of abundant NiO–CeO_2_ active interfaces, effectively inducing surface Ce^3+^ species and oxygen vacancies. These merits significantly enhanced the reactor’s oxygen adsorption capacity and redox properties, thus realizing efficient methane activation in catalytic oxidation. Moreover, the optimal reactor successfully passed 10 thermal cycle tests, further verifying the thermal stability of the catalytic structure. In addition, it exhibited outstanding long-term stability during a 100 h test, with no carbon deposition or active phase sintering observed. This work develops an optimized straight-channel NiO/CeO_2_ ceramic reactor and offers a practical and scalable design strategy for VAM oxidation.

## 1. Introduction

Methane is an important greenhouse gas with a global warming potential (GWP) 28–34 times higher than CO_2_ over a 100-year period, making its emission control critical for mitigating near-term climate change [1,2]. Among the various CH_4_ emission sources, coal mine ventilation air methane (VAM) is one of the major anthropogenic sources, accounting for 8–10% of global methane emissions [3]. However, the low methane concentration (0.1–1.5 vol%) in VAM not only precludes direct thermal combustion but also renders physical separation technologies economically prohibitive for large-scale application [4,5]. Consequently, the cost-effective and environmentally friendly abatement and utilization of VAM remains a significant technological challenge in the coal industry and environmental field.

Catalytic oxidation of VAM enables operation at temperatures below 650 °C, significantly reducing energy consumption and secondary pollutant emissions compared to conventional thermal oxidation (above 900 °C) [6,7,8,9]. The performance of this technology hinges on the development of high-efficiency catalysts and structurally optimized reactors. Precious metal catalysts (Pd, Pt) exhibit excellent low-temperature catalytic activity but are constrained by high costs and sintering-induced deactivation during long-term operation [10,11,12,13,14]. Although perovskite-type oxides demonstrate good structural stability, they suffer from low efficiency in activating the strong C-H bonds of methane [15,16,17,18,19]. Non-noble metal oxides, particularly NiO, attract significant attention due to their superior C-H bond activation capability and low cost [20,21]. CeO_2_ utilizes its Ce^3+^/Ce^4+^ redox cycle to enable efficient oxygen storage and release [22,23]. NiO/CeO_2_ composite materials exhibit excellent catalytic performance for methane oxidation due to their unique synergistic interaction mechanism at the heterointerface [24,25,26]. However, most existing studies focused on powdered NiO/CeO_2_ catalysts, which suffer from inherent drawbacks such as localized heat accumulation, active phase sintering, and limited mass transfer efficiency; structured catalysts are usually prepared via complex impregnation or coating processes, which easily lead to the non-uniform distribution of active phases and weak adhesion, making it difficult to balance catalytic performance and structural stability [27,28].

Structured ceramic reactors that integrate thermal storage and catalytic functions present a promising solution to these challenges. Nevertheless, traditional fabrication techniques such as impregnation or coating often result in non-uniform distribution of the active phase and weak adhesion [29]. Fortunately, the direct co-sintering of NiO and CeO_2_-based ceramic provides the dual benefits of high catalytic activity and enhanced structural stability, as demonstrated in solid oxide cell (SOC) electrodes [30,31,32,33]. However, the mass transfer efficiency of VAM within the conventional ceramic reactor is severely limited by the sponge-like disordered pores. An effective way to address mass transfer limitations is to develop straight channels in ceramic reactors. The mesh-assisted phase inversion enabled the fabrication of ceramics with graded straight-channel structures, which can realize integrated molding of the reactor in one step, and thus provide simultaneous ideal mass transfer and highly efficient catalytic performance [34,35,36]. This structural characteristic was demonstrated in SOCs, which achieved not only the utilization of low-concentration fuels, but also the efficient reforming of various hydrocarbons, including methane, ethanol, and coal mine methane [37,38,39].

**Figure 1:**
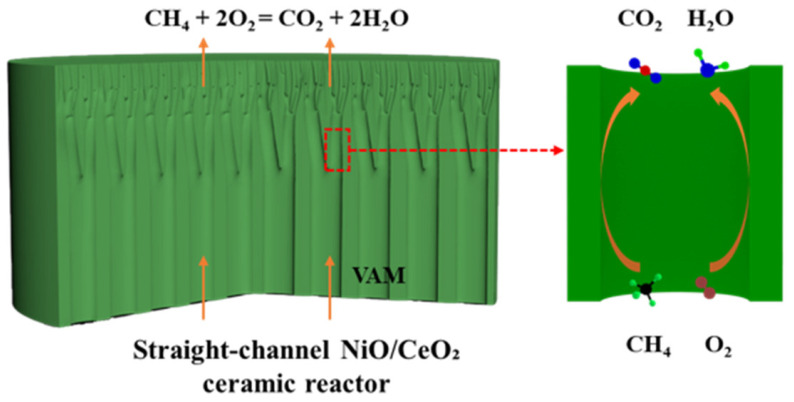
Schematic of the straight-channel NiO/CeO_2_ ceramic reactor for the catalytic oxidation of VAM.

In this study, a NiO/CeO_2_ ceramic reactor with a straight-channel was prepared via mesh-assisted phase inversion for VAM catalytic oxidation [40]. As shown in Figure 1, VAM was efficiently catalytically oxidized into CO_2_ and H_2_O while being transported through the reactor channels. This one-step preparation method realizes the uniform dispersion of active components and precise regulation of the straight-channel structure, effectively solving the problems of poor active phase adhesion and low mass transfer efficiency in traditional structured catalysts. The reactor with 60 wt% NiO content achieved a methane conversion rate of 95% at 650 °C, along with excellent operational stability. This work offers a promising technical method for the efficient and stable combustion of VAM.

## 2. Materials and Methods

### 2.1. Materials

NiO (≥99%) and CeO_2_ (≥99%) powders were used as ceramic precursors. Polyvinylpyrrolidone (PVP 40000) and polyethersulfone (PES) served as polymer binders, and N-methylpyrrolidone (NMP) was used as the solvent. All reagents were obtained from commercial sources and used directly.

### 2.2. Fabrication of Straight-Channel NiO/CeO_2_ Ceramic Reactors

**Figure 2:**
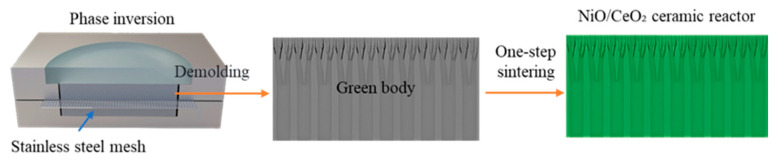
Schematic diagram of the fabrication process for the NiO/CeO_2_ ceramic reactor via mesh-assisted phase inversion.

As shown in Figure 2, straight-channel NiO/CeO_2_ ceramic reactors were fabricated via a mesh-assisted phase inversion. This method for preparing straight channel ceramics was reported in numerous studies [32,41,42,43]. A precursor solution was first prepared by dissolving polyethersulfone (PES, 4.59 wt%) and polyvinylpyrrolidone (PVP 40000, 0.47 wt%) in N-methylpyrrolidone (NMP, 25.94 wt%). Ceramic powders (NiO and CeO_2_) with NiO mass fractions of 20, 50, 60, and 70 wt% were then mixed into the solution to form the phase inversion slurries, with a constant ceramic powder loading of 69.0 wt%. The green body was formed in a custom mold and subjected to phase inversion for 2 h. It was then immersed in deionized water for 12 h to remove residual solvent, followed by drying at 60 °C for 12 h. Finally, sintering was conducted at 1270 °C, 1300 °C, or 1330 °C for 5 h to produce the final reactor. The mechanical properties of the reactors were tested by a YC-5M microcomputer-controlled bending testing machine (Jinan Yongce, Jinan, China) including compressive strength to evaluate structural robustness; thermal shock resistance tests were carried out with the protocol of holding at 650 °C for 1 h followed by cooling to room temperature at a rate of 5 °C min^−1^ for 10 cycles.

### 2.3. Characterization

Crystalline phases were analyzed by X-ray diffraction (XRD, Bruker D8 ADVANCE, Bruker Corp., Billerica, MA, USA) with Cu Kα radiation. The crystallite sizes of the samples were calculated using the Scherrer equation to further analyze the structural evolution of NiO/CeO_2_ composites with different NiO contents. Morphology and microstructure were examined using scanning electron microscopy (SEM, ZEISS, Carl Zeiss AG, Oberkochen, Germany). Brunauer–Emmett–Teller (BET) specific surface area measurement was performed via N_2_ adsorption–desorption at −195.850 °C on a ASAP 2020 Plus HD88 surface area and porosity analyzer (Micromeritics Instrument Corp., Norcross, GA, USA). The sample was degassed at 30 °C for 10 min prior to testing. H_2_-temperature-programmed reduction (H_2_-TPR) was conducted on a VDSorb-91i analyzer (Vodo Instrument Corp., Guangzhou, China) equipped with a thermal conductivity detector. Surface elemental composition and chemical states were analyzed by X-ray photoelectron spectroscopy (XPS, ESCALAB 250XI, Thermo Fisher Scientific Inc., Waltham, MA, USA) with an Al Kα source. All XPS binding energies were calibrated against the C 1s peak at 284.8 eV.

### 2.4. Catalytic Activity

The straight-channel ceramic reactor, with a geometric diameter of 16 mm and a thickness of 1 mm (corresponding to a catalyst volume of ~0.201 cm^3^), was positioned within a quartz tube, packed and fixed in place with quartz wool, and then heated to the target temperature in a tube furnace at a heating rate of 5 °C min^−1^. A simulated VAM mixture was then introduced into the reactor. To systematically investigate the effects of key parameters, the methane concentration in the feed was varied at 2, 3, and 4 vol% while maintaining oxygen at 20 vol%, with the balance being N_2_. The total gas flow rates were set at 20, 40 and 60 sccm, corresponding to gas hourly space velocities (GHSV) of approximately 5970, 11,940 and 17,910 h^−1^, to examine the influence of space velocity. In addition, steam was introduced into the feed stream at concentrations of 3 and 10 vol% using a custom-built steam generator to evaluate its impact on the catalytic performance. The effluent gas composition was analyzed online with a gas chromatograph (Shimadzu GC-2014, Shimadzu Corp., Kyoto, Japan). All core catalytic performance tests were conducted with three independent repetitive experiments to ensure reproducibility. Experimental errors were calculated based on the standard deviation (SD). Error bars representing SD were added to all catalytic performance curves. Methane conversion (*η*) was calculated according to the following equation:

$$\eta =\frac{{\left[{\mathrm{CH}}_{4}\right]}_{\mathrm{in}}-{\left[{\mathrm{CH}}_{4}\right]}_{\mathrm{out}}}{{\left[{\mathrm{CH}}_{4}\right]}_{\mathrm{in}}}\times 100\%$$

where [CH_4_]_out_ and [CH_4_]_in_ denote the inlet and outlet methane concentrations, respectively.

## 3. Results

### 3.1. Effect of Ceramic Composition

**Figure 3:**
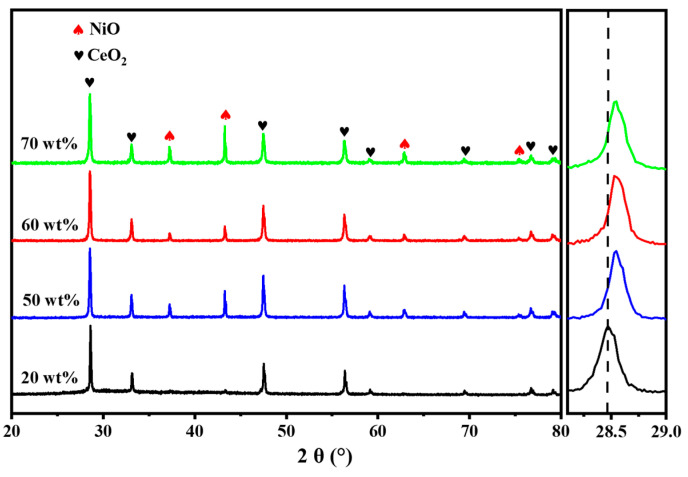
XRD patterns of NiO/CeO_2_ ceramic reactors with different NiO contents.

The active sites of NiO/CeO_2_ ceramic microreactors were governed by the NiO content, which significantly influenced their catalytic oxidation performance toward VAM [9,44]. Therefore, ceramic microchannel reactors with NiO mass fractions of 20%, 50%, 60%, and 70% were fabricated in this study. All reactors were sintered at 1300 °C. XRD analysis confirmed the formation of a two-phase composite structure comprising NiO and CeO_2_ in all samples (Figure 3). With increasing NiO content, the NiO diffraction peaks gradually intensified and shifted slightly toward higher angles. This shift was primarily attributed to lattice contraction induced by the incorporation of Ni^2+^ into the CeO_2_ lattice. The grain size of the NiO phase in NiO–CeO_2_ composite ceramics with different NiO contents was calculated by broadening analysis of the characteristic diffraction peaks of NiO using the Scherrer equation. The results show that with increasing NiO content, the average grain size of NiO increases gradually, reaching approximately 165 nm at 20 wt%, 195 nm at 50 wt%, 220 nm at 60 wt%, and 255 nm at 70 wt%. This size evolution can be attributed to the higher NiO content providing a greater driving force for grain boundary migration during the one-step ceramic sintering process, thereby promoting moderate grain growth of NiO [45]. Furthermore, no additional impurity phases were detected in any of the samples, indicating good chemical compatibility between NiO and CeO_2_.

**Figure 4:**
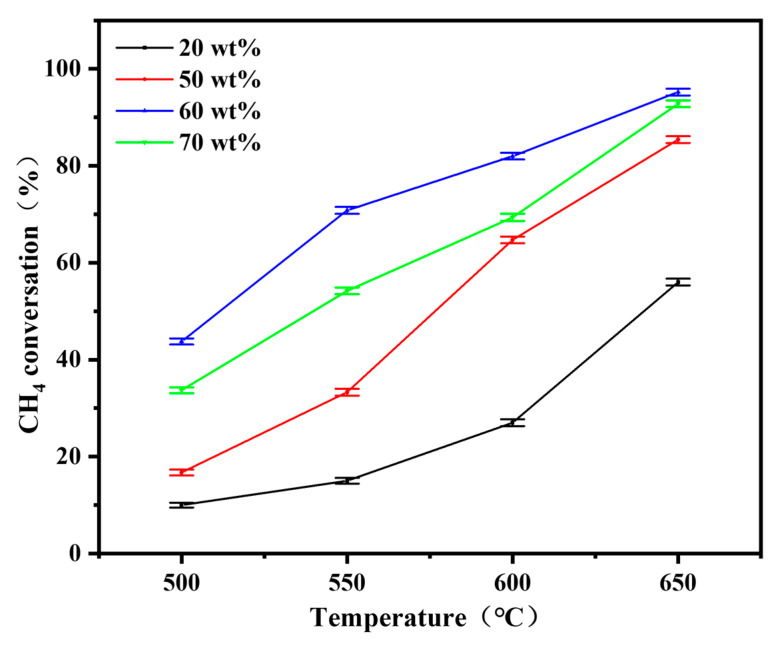
Catalytic VAM performance of reactors with different NiO contents.

The catalytic performance was evaluated under simulated VAM conditions (2% CH_4_, 20 sccm). Catalytic performance tests revealed that both the NiO content and operating temperature significantly affected the methane oxidation activity [46]. As shown in Figure 4, methane conversion generally increased with operation temperature across all compositions. The 60 wt% NiO reactor exhibited superior catalytic performance, achieving 95% methane conversion at 650 °C, which was significantly higher than that of reactors with 70 wt% (92%), 50 wt% (85.4%), and 20 wt% (56%) NiO content. The composite of NiO and CeO_2_ forms highly reactive heterointerfaces that serve as the core active sites for the methane oxidation reaction [25,26]. NiO acts as the active center to cleave the strong C-H bonds of methane molecules while CeO_2_ regulates the adsorption and activation of oxygen species through its Ce^3+^/Ce^4+^ redox cycle at the interface, with the synergistic catalytic effect between the two phases being highly dependent on the number of effective heterointerfaces formed [20,22]. The 60 wt% NiO content reached the optimal component ratio to maximize the construction of such active heterointerfaces. Back-scattered scanning electron microscopy (BSE-SEM) results (Figure S1) confirm the uniform dispersion of NiO and CeO_2_ phases in this optimal sample, which not only builds abundant NiO–CeO_2_ heterointerfaces, but also guarantees the sufficient exposure of active sites. Meanwhile, BET specific surface area tests revealed that the 60 wt% NiO reactor had the highest surface area (4.55 m^2^ g^−1^) compared to 3.05, 3.73, and 4.25 m^2^ g^−1^ for the 20, 50, and 70 wt% NiO reactors, respectively. This maximum specific surface area further optimizes the gas–solid mass transfer efficiency during the catalytic reaction. Combined with the moderate NiO crystallite size regulated by this optimal component ratio, the above merits synergistically boosted the intrinsic catalytic activity and mass transfer performance of the reactor, thereby realizing the optimal methane oxidation efficiency [44,47].

**Figure 5:**
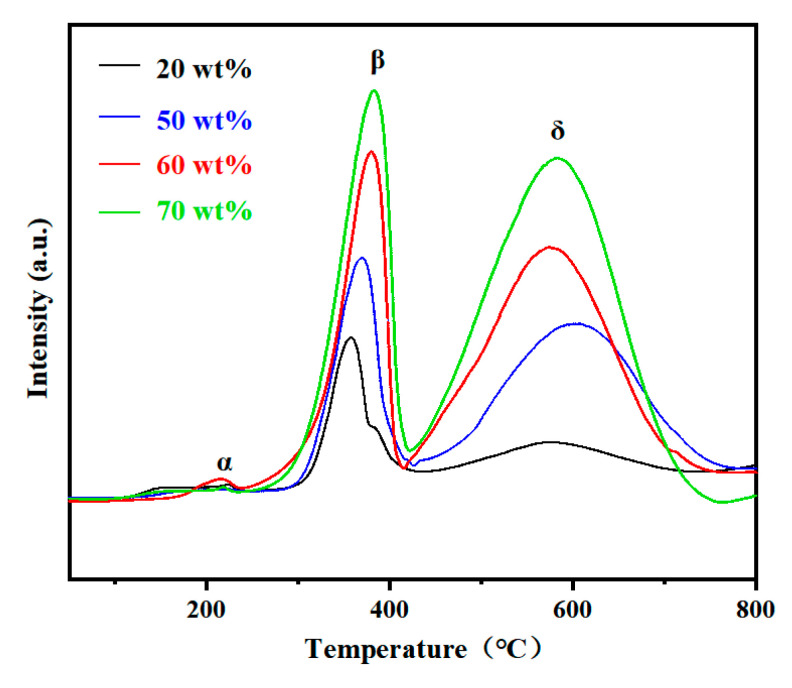
H_2_-TPR profiles of reactors with different NiO contents.

H_2_-TPR was conducted to evaluate the reducibility of the NiO/CeO_2_ reactors (Figure 5). Three reduction peaks were observed for all reactors, with the α peak corresponding to the reduction in surface adsorbed oxygen species, the β peak to the reduction in agglomerated NiO species, and the δ peak to the reduction in bulk CeO_2_ lattice oxygen, respectively [24,46]. With increasing NiO content, the intensities of both the β and δ peaks gradually increased, indicating a rise in active interfaces and reducible oxygen species in the composite system [23]. Notably, the reduction temperature of the δ phase reached a relatively low level at 60 wt% NiO, which was attributed to the optimized NiO–CeO_2_ interface formed at this proportion that promotes hydrogen spillover between the two phases, thus effectively lowering the reduction temperature of CeO_2_ lattice oxygen [26,46]. The enhanced reducibility of lattice oxygen at the optimal NiO content further verifies the strong synergistic interaction between NiO and CeO_2_, which is conducive to the continuous supply of active oxygen species for the methane oxidation reaction and thus further improves the catalytic performance of the reactor [21].

**Figure 6:**
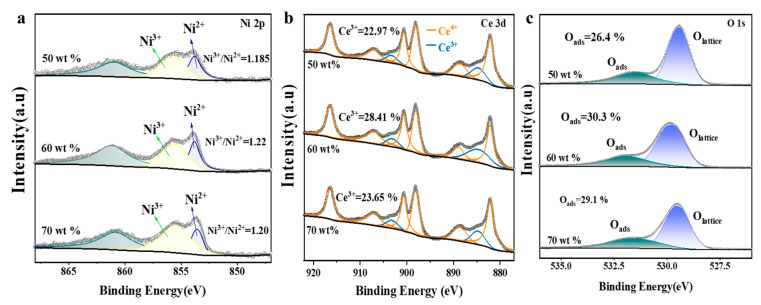
XPS spectra of reactors with different NiO contents: (**a**) Ni 2p; (**b**) Ce 3d; (**c**) O 1s.

XPS analysis further revealed the surface chemical structure characteristics. The 60 wt% NiO/CeO_2_ reactor exhibited the highest Ni^3+^/Ni^2+^ ratio (Figure 6a), confirming stronger electronic interaction with CeO_2_. Its Ce 3d spectrum (Figure 6b) showed the highest surface Ce^3+^ concentration (28.41%), with the most pronounced characteristic peaks at ~881.5 and ~899.0 eV, indicating that an appropriate NiO/CeO_2_ composite ratio facilitated the formation of more oxygen vacancies. As shown in Figure 6c, it also displayed the highest proportion of active adsorbed oxygen (Oads, 30.3%). Therefore, the strong electronic interaction between Ni^2+^ and CeO_2_ in this reactor promoted the generation of surface Ce^3+^ and oxygen vacancies, thereby enhancing oxygen adsorption and activation capability and constructing a more favorable redox reaction interface [22,25].

### 3.2. Effect of Sintering Temperature

**Figure 7:**
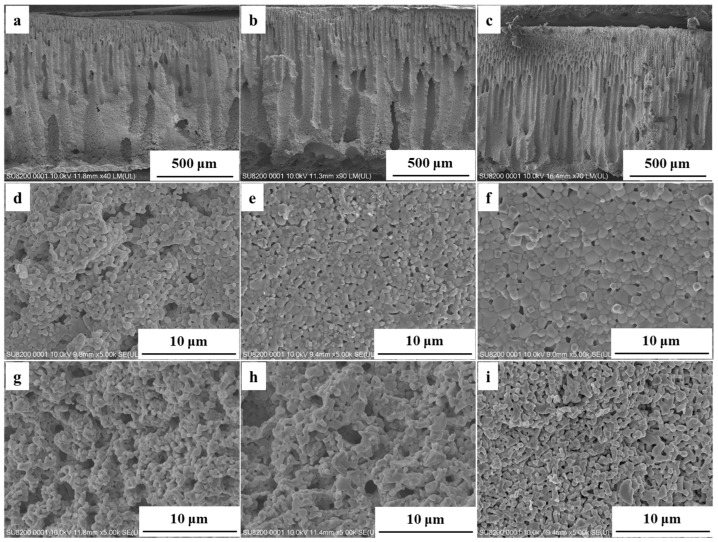
SEM images of the ceramic reactor’s microstructure after sintering at (**a**,**d**,**g**) 1270 °C, (**b**,**e**,**h**) 1300 °C, and (**c**,**f**,**i**) 1330 °C. Cross-section (**a**–**c**), skin layer (**d**–**f**), and pore-wall (**g**–**i**).

**Figure 8:**
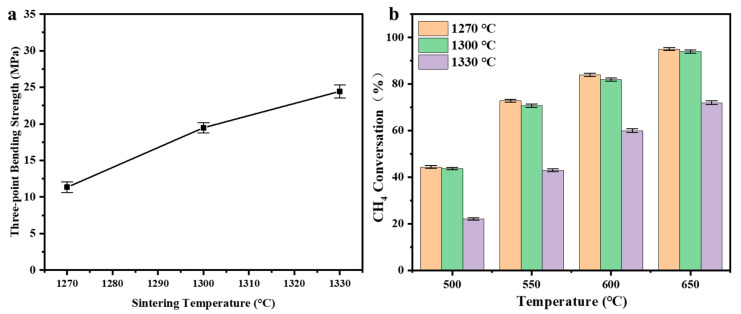
Effect of sintering temperature on (**a**) compressive strength and (**b**) catalytic VAM oxidation performance.

In this study, NiO/CeO_2_ ceramic reactors sintered at 1270 °C, 1300 °C, and 1330 °C were compared to analyze the effect of sintering temperature on their microstructure and catalytic performance. As the sintering temperature increased from 1270 °C to 1330 °C, SEM showed a gradual decrease in cross-sectional pore size (Figure 7a–c), a continuous decline in surface porosity accompanied by obvious grain coarsening (Figure 7d–f) and significant densification of the pore-wall structure (Figure 7g–i). Grain coarsening reduced the number of active interfaces, thereby weakened the intrinsic catalytic activity [25]. BET tests showed that the specific surface area of the reactor decreased from 5.12 m^2^ g^−1^ (1270 °C) to 4.55 m^2^ g^−1^ (1300 °C) and further to 3.72 m^2^ g^−1^ (1330 °C) with increasing sintering temperature. As shown in Figure 8a, mechanical property tests showed that the reactors sintered at 1270, 1300, and 1330 °C exhibited compressive strengths of 11.34, 19.47, and 24.45 MPa, respectively. The increase in sintering temperature significantly improved the mechanical strength of the reactor, which was attributed to the densification of the pore-wall structure and enhanced bonding between ceramic particles during high-temperature sintering. As shown in Figure 8b, methane conversion gradually decreased with increasing sintering temperature. At a reaction temperature of 650 °C, when the sintering temperature was raised from 1270 °C to 1300 °C, methane conversion only slightly decreased from 96.5% to 95.2%, indicating that the reactor maintained excellent catalytic activity. However, when the temperature was further increased to 1330 °C, the conversion dropped significantly to 72.6%. Increasing the sintering temperature appropriately promoted the bonding of ceramic particles and densification of the material, thereby improving mechanical strength and structural stability. However, excessive sintering induced pronounced grain coarsening, leading to a significant decrease in catalytic activity [45]. Therefore, based on the balance between catalytic activity, specific surface area, and mechanical integrity from microstructure densification at elevated temperatures, 1300 °C was determined as the optimal sintering temperature at which the reactor achieves both excellent catalytic performance and sufficient mechanical robustness for practical application.

### 3.3. Effect of Operating Conditions

**Figure 9:**
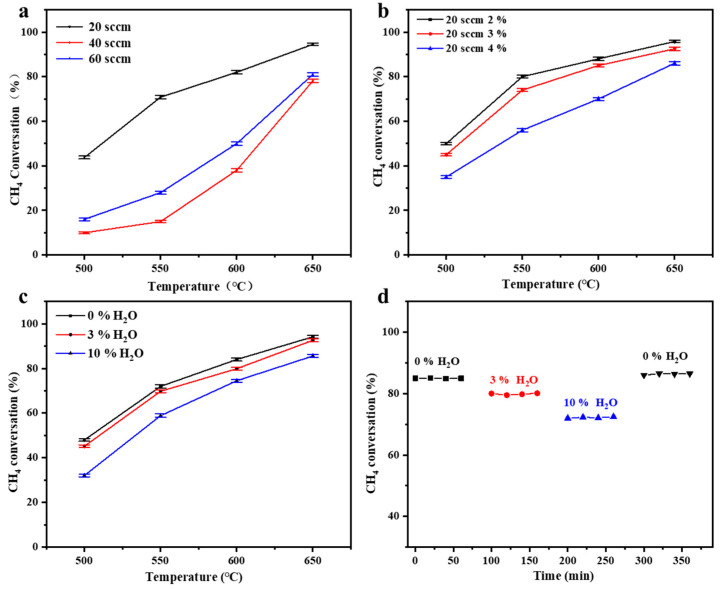
CH_4_ conversion under varying operational conditions: (**a**) VAM flow rate; (**b**) CH_4_ concentration; (**c**) effect of steam content on stability of catalyst performance; (**d**) effect of steam on the stability of methane conversion at 600 °C and 30 sccm.

The performance of VAM catalytic oxidation was influenced by key operating parameters, including the gas flow rate, methane concentration, and steam, which affected the reaction through the regulation of both the mass transfer-reaction equilibrium and the competitive adsorption behavior on the catalyst surface. As shown in Figure 9a, the CH_4_ conversion decreased with increasing gas flow rate, a trend that was more pronounced at lower temperatures. For example, at a methane concentration of 2%, when the flow rate was increased from 20 sccm to 60 sccm, the conversion decreased from 95% to 78% at 650 °C and dropped sharply from 82% to 38% at 600 °C. This change was attributed to the reduction in reactant residence time caused by the higher flow rate, which made intrapore diffusion mass transfer within the microchannels the rate-limiting step [37,42]. Similarly, an increase in methane concentration also led to a decline in conversion (Figure 9b). At a flow rate of 20 sccm and 600 °C, the methane conversions were 88.2%, 82.4%, and 75.6% at methane concentrations of 2%, 3%, and 4%, respectively. This phenomenon was primarily due to the increased reactant flux, while the number of active sites on the catalyst surface remained limited, imposing intrinsic kinetic constraints on the reaction [7,47]. To simulate real VAM conditions, the effect of steam was further investigated. The results showed that steam exhibited a clear inhibitory effect on methane oxidation, and the extent of inhibition increased with higher steam concentration (Figure 9c). Although the conversion gradually declined after steam introduction, the reactor maintained structural stability under continuous steam exposure at 600 °C and 30 sccm (Figure 9d). After stopping the steam supply, the activity was promptly recovered, thus verifying that the inhibitory effect of steam was completely reversible. This reversibility mainly stems from the competitive adsorption of water molecules on the active sites, without causing sintering or structural damage to the active phase [27].

### 3.4. Durability

**Figure 10:**
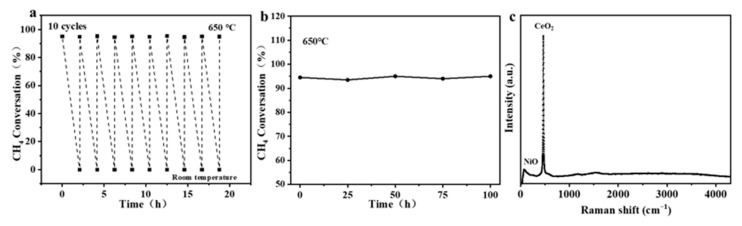
(**a**) Thermal cycling stability and (**b**) post-test long-term stability of the ceramic reactor for VAM catalysis, accompanied by (**c**) corresponding Raman spectroscopy results.

**Figure 11:**
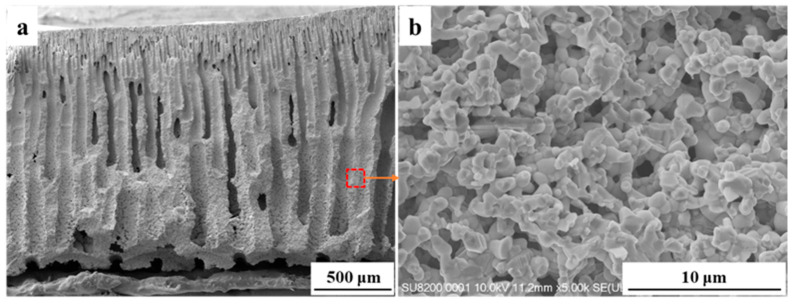
SEM images of the reactor after stability test: (**a**) overall cross-sectional structure; (**b**) magnified morphology.

Durability is a critical parameter for evaluating the practical application potential of the reactor. As shown in Figure 10a, 10 thermal cycle tests were conducted to evaluate the thermal stability of the catalytic structure, with the protocol of holding at 650 °C for 1 h followed by cooling to room temperature at a rate of 5 °C min^−1^. The results indicated that the methane conversion rate remained above 93% after 10 thermal cycles with almost no performance attenuation and no periodic fluctuations, confirming that the catalytic structure maintained stable catalytic performance under thermal shock conditions involving repeated switching between high and low temperatures. SEM characterization after thermal cycles showed that the straight-channel structure remained intact with no obvious changes in the micromorphology of the pore walls and grains, directly verifying the excellent thermal shock resistance of the reactor. The durability of the 60 wt% NiO/CeO_2_ ceramic reactor was tested at 650 °C with a feed of 3% CH_4_ at a flow rate of 20 sccm for 100 h. As shown in Figure 10b, the reactor achieved a methane conversion of about 95% with fluctuations below 3%. After the test, Raman spectroscopy and SEM were employed to characterize the microstructure of the reactor. Raman spectroscopy revealed no characteristic carbon peaks (typically around 1350 cm^−1^ and 1580 cm^−1^), indicating the absence of carbon deposition on the reactor surface (Figure 10c). SEM images showed that the straight-channel structure remained intact, and no significant sintering or agglomeration of NiO and CeO_2_ particles was observed, confirming its robust structural stability (Figure 11). This is attributed to the straight-channel design, which effectively prevents the formation of localized hot spots and inhibits active phase sintering. Meanwhile, the NiO–CeO_2_ interfacial interaction stabilizes the active species and promotes the mobility and replenishment of lattice oxygen, thereby suppressing carbon deposition. Therefore, the reactor exhibited strong potential for practical applications.

## 4. Discussion

In this study, a straight-channel NiO/CeO_2_ ceramic reactor was fabricated via a one-step mesh-assisted phase-inversion process, enabling the efficient catalytic oxidation of VAM. The catalyst is thus directly integrated into a monolithic straight-pore ceramic framework, providing the reactor with a robustness and uniformity that obviate the low mass transfer efficiency, uneven active phase distribution, and poor stability characteristic of conventional packed-bed or coated systems.

The optimal NiO loading was 60 wt%, at which abundant NiO–CeO_2_ heterointerfaces were formed and the highest concentrations of Ce^3+^ and oxygen vacancies were induced, thereby significantly enhancing oxygen adsorption and activation as well as the efficiency of C-H bond cleavage. Correspondingly, this loading yielded the largest specific surface area and a methane conversion of 95% at 650 °C. Sintering temperature exhibited a trade-off between mechanical strength and catalytic activity, as higher temperatures boost strength while inhibiting catalytic performance. 1300 °C was identified as the optimal balance point, providing adequate mechanical strength while maintaining high catalytic activity. Operating conditions such as flow rate, methane concentration, and steam affected conversion via shifts in the mass-transfer-reaction balance or competitive adsorption. Steam only induced reversible competitive adsorption, and activity returned quickly after its removal. Therefore, this reactor is well-suited for practical operating conditions of VAM treatment. Durability tests confirmed its practical potential. After 10 thermal cycles, methane conversion remained above 93%, and during 100 h long-term operation, it stabilized around 95%, with no carbon deposition or active phase agglomeration. This benefits from the excellent mass and heat transfer capability of the straight-channel structure, as well as the stabilizing effect brought by the ceramic anchoring of the NiO–CeO_2_ interface.

The reactor fabricated by the one-step mesh-assisted phase inversion method features both a straight pore-catalyst coupled structure and a uniform distribution of active phases. It effectively overcomes the drawbacks of poor mass transfer in conventional porous ceramics and weak adhesion and non-uniform distribution inherent to traditional impregnation and coating methods. The construction of this reactor not only presents a new technical approach for VAM treatment and resource recovery, but also sheds light on the development of structured ceramic catalytic reactors for environmental catalysis.

## Data Availability

The original contributions presented in this study are included in the article/Supplementary Material. Further inquiries can be directed to the corresponding authors.

## Supplementary Materials

The following supporting information can be downloaded at: https://www.mdpi.com/article/10.3390/ma19091718/s1, Figure S1: Morphology and phase dispersion of the 60 wt% NiO/CeO_2_ ceramic reactor sintered at 1300 °C.
