# Supplementary material for: Straight-Channel NiO/CeO2 Ceramic Reactor Fabricated via Mesh-Assisted Phase Inversion for Catalytic Oxidation of Ventilation Air Methane
Source: Materials (Basel). 2026 Apr 23;19(9):1718. doi: 10.3390/ma19091718 (PMC13164882; doi:10.3390/ma19091718)
Supplement: Supplementary file 1 [file materials-19-01718-s001.zip › materials-4242280-supplementary.pdf]

Supporting Information

Straight-Channel NiO/CeO_2 Ceramic Reactor Fabricated via Mesh-Assisted Phase Inversion for Catalytic Oxidation of Ventilation Air Methane

Fangsheng Liu [1,*], Enming Shi [1], Zhiqiang Cao [1], Xuemei Ou [1], Fangjun Jin [1], Dingying Zhou [1], Zhen Wang [1], Xinyi Han [1], Shiru Le [2], Yeqing Wang [1,*]

[1] School of Materials Science and Physics, China University of Mining and Technology, Xuzhou 221116, China; tbh357@cumt.edu.cn; 648194451@qq.com; caozq6666@163.com; oxm@cumt.edu.cn; jinfj@cumt.edu.cn; zdy051010@163.com; wangzhen25@cumt.edu.cn; hhhhxxxyyy111@163.com; yeqingwang@cumt.edu.cn

[2] School of chemistry and chemical engineering, Harbin Institute of Technology, Harbin, Heilongjiang 150001, People's Republic of China; leshiru@hit.edu.cn

* Correspondence: yeqingwang@cumt.edu.cn; tbh357@cumt.edu.cn (Y.W. and F.L.)

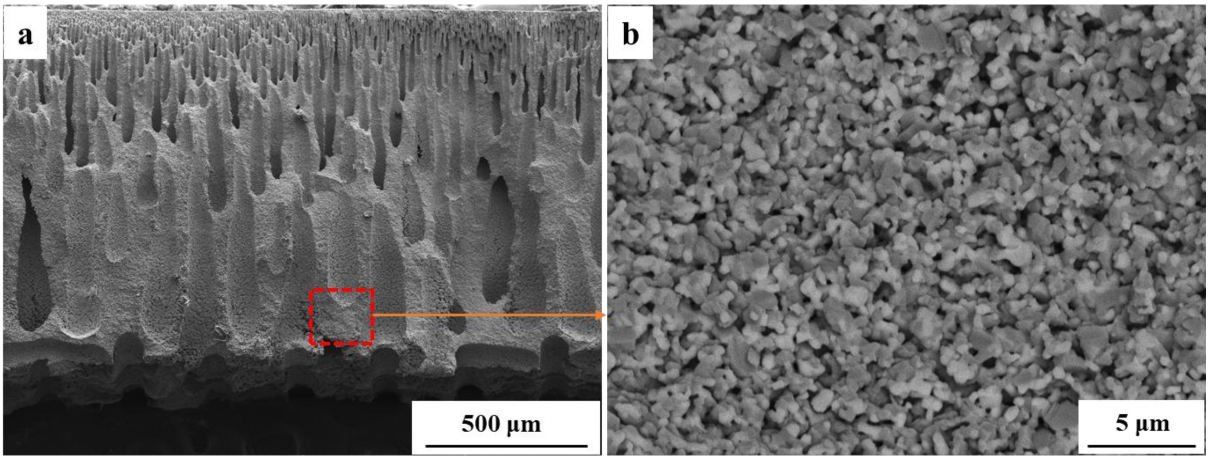

Figure S1. Morphology and phase dispersion of the 60 wt% NiO/CeO_2 ceramic reactor sintered at 1300 °C. (a) Cross-sectional SEM image; (b) BSE-SEM image, where bright and dark regions correspond to NiO and CeO_2 phases, respectively.
